# *Providencia vermicola* Infection Alters Bacterial and Microeukaryotic Gut Community Composition in Nile Tilapia

**DOI:** 10.3390/ani16081180

**Published:** 2026-04-12

**Authors:** Jesús Salvador Olivier Guirado-Flores, Francisco Vargas-Albores, Marcel Martínez-Porchas, Estefanía Garibay-Valdez, Diana Medina-Félix, Luis Rafael Martínez-Córdova, Francesco Cicala, Pablo Martinez-Lara

**Affiliations:** 1 Centro de Investigación en Alimentación y Desarrollo, Hermosillo 83304, Mexico; jguirado223@estudiantes.ciad.mx (J.S.O.G.-F.); fvalbores@ciad.mx (F.V.-A.); estefania.garibay@ciad.mx (E.G.-V.); diana.medina@ues.mx (D.M.-F.); 2 Departamento de Ecología, Universidad Estatal de Sonora, Hermosillo 83100, Mexico; 3 Departamento de Investigaciones Científicas y Tecnológicas de la Universidad de Sonora, Universidad de Sonora, Hermosillo 83000, Mexico; luis.martinez@unison.mx; 4 Water Research Institute, National Research Council, 28922 Verbania, Italy; fracicala@gmail.com; 5 Instituto de Acuacultura del Estado de Sonora, Hermosillo 83280, Mexico; linkathome@hotmail.com

**Keywords:** Nile tilapia, *Providencia vermicola*, gut microbiota, bacterial community, microeukaryotes

## Abstract

Nile tilapia (*Oreochromis niloticus*) is a major aquaculture species worldwide, yet bacterial infections remain a critical constraint to production sustainability. Although pathogen-associated dysbiosis has been widely described, most studies have focused exclusively on bacterial communities, overlooking the multi-kingdom nature of the intestinal microbiota. This study evaluated the impact of experimental *Providencia vermicola* infection on both prokaryotic and microeukaryotic intestinal communities under controlled conditions. Using 16S (V3–V4) and 18S (V9) rRNA amplicon sequencing, we compared healthy and infected fish and assessed taxonomic, structural, and predicted functional changes. Infection was associated with significant compositional shifts, including increased relative abundances of Bacteroidota and Proteobacteria and decreased relative abundances of Fusobacteriota and Patescibacteria. Concomitantly, microeukaryotic groups such as Protalveolata, Nematozoa, and Phragmoplastophyta were significantly reduced. Functional prediction revealed metabolic pathway reconfiguration consistent with infection-associated ecological disturbance. Together, these results suggest that the pathogen challenge is associated with coordinated changes in the intestinal microbiota as an integrated system across multiple microbial kingdoms rather than as isolated bacterial shifts. This study supports ecosystem-level interpretations of dysbiosis and highlights the importance of incorporating cross-domain analyses into health assessment strategies in aquaculture species.

## Simple Summary

Nile tilapia is one of the most widely farmed fish species worldwide, but infectious outbreaks remain a key challenge for sustainable aquaculture. Bacterial infections are known to disrupt gut microbiota, a microbial community essential for digestion, immunity, and overall health. However, most studies focus only on bacteria, ignoring other microorganisms that coexist in the intestine. In this study, we experimentally infected tilapia with *Providencia vermicola* via intraperitoneal injection and analyzed both bacterial and eukaryotic intestinal communities to obtain a more comprehensive view of gut ecosystem responses. Infection led to coordinated shifts across microbial kingdoms, including the expansion of opportunistic bacteria and the reduction of specific microeukaryotic groups. These findings suggest that disease affects the intestinal microbiota as an integrated ecosystem rather than through isolated bacterial changes. Understanding these multi-kingdom responses may improve health monitoring and management strategies in aquaculture systems.

## 1. Introduction

Nile tilapia, *Oreochromis niloticus* (Linnaeus, 1758), is one of the most important freshwater species for global aquaculture due to its high production and relevance to global food security. According to FAO statistics, global production of Nile tilapia reached approximately 5.3 million tons in 2022, including about 5.0 million tons from aquaculture and 0.3 million tons from capture fisheries, positioning it among the most widely farmed fish worldwide [1]. Its rapid growth, tolerance to diverse environmental conditions, and ability to adapt to intensive farming systems have favored its expansion in modern aquaculture [2,3].

However, intensive aquaculture systems may expose fish to multiple stressors, such as fluctuations in water quality or inadequate diets, leading to physiological changes that can disrupt the stability of the intestinal microbiota. These disturbances may favor the establishment of pathogenic bacteria and contribute to the development of infectious diseases [4,5]. In this context, the intestine is recognized as a principal route of infection, facilitating successful pathogen colonization and the progression of pathological processes in the host [6,7].

Among emerging pathogens, species of the genus *Providencia*, belonging to the order Enterobacterales, have been associated with diseases in tilapia. Specifically, *Providencia vermicola* has been reported to cause clinical signs such as exophthalmia, fin and ocular ulcerations, and anorexia [8,9].

Fish health is closely linked to the intestinal microbiota, which plays essential roles in maintaining the intestinal barrier, modulating the immune system, and preventing the overgrowth of opportunistic microorganisms [10,11,12]. This microbiota comprises a diverse assemblage of microorganisms, including bacteria, archaea, viruses, fungi, and protozoa [13], which dynamically interact with the host to maintain intestinal homeostasis.

Bacterial infections can significantly alter the composition of the intestinal microbiota, affecting key processes such as energy metabolism, immune responses, and microbial balance in the host [14]. Although the relationship between intestinal microbiota and host health has been widely studied in humans and other mammals [15,16,17], information on these processes in fish remains limited, particularly regarding intestinal eukaryotic microorganisms in tilapia.

We hypothesized that *P. vermicola* infection would not only alter bacterial composition but also disrupt the coordinated structure of the intestinal microbiota across microbial kingdoms, leading to a detectable shift in community organization.

In this study, prokaryotic and microeukaryotic intestinal microbial communities of healthy Nile tilapia and fish experimentally infected with *P. vermicola* were compared. The composition of the intestinal microbiota in healthy and diseased Nile tilapia was investigated using sequencing-by-synthesis, targeting the V3–V4 and V9 regions of the 16S rRNA and 18S rRNA genes, respectively.

## 2. Materials and Methods

### 2.1. Experimental Design

Juvenile female Nile tilapia (*O. niloticus*), certified as pathogen-free, were obtained from the Aquaculture Center of the State of Sonora (CAES), located in Ciudad Obregón, Sonora, Mexico, and transported to the Wet Laboratory of the Department of Scientific and Technological Research at the University of Sonora, in Hermosillo, Sonora, where the experimental trial was conducted. Fish were acclimated for 15 days in freshwater at 20 ± 0.5 °C and fed twice daily with a commercial diet (Silver Cup^®^, 2.5 mm pellets, 45% protein and 16% lipid; El Pedregal, Toluca, México). Prior to the experiment, the fish showed no visible lesions and exhibited regular swimming and feeding behavior, suggesting good health. All experimental procedures were performed in accordance with ethical guidelines approved by the CIAD Ethics Committee (Registration: CONBIOÉTICA-26-CEI-001-20200122).

The experimental units, also referred to as tanks, were 50 L aquaria with an operational volume of 35 L of freshwater, continuously aerated (>6 mg/L), and at a constant temperature of 20 ± 0.5 °C under a 12 h light:12 h dark photoperiod. Water quality was monitored daily using commercial kits and an aquaculture photometer (HI 83293, Hanna Instruments, Woonsocket, RI, USA), including measurements of ammonia (NH_4_^+^), nitrite (NO_2_^−^), and nitrate (NO_3_^−^). The water quality analysis results are provided in Supplementary Table S1.

Fish were randomly assigned to two experimental treatments (*n* = 24 fish per treatment): a control group inoculated with saline solution and an infected group inoculated with *P. vermicola*. Each treatment was performed in triplicate, with eight fish per tank (mean initial weight: 21.3 ± 2.52 g), and each tank was considered an independent experimental replicate. Briefly, for microbiota analyses, the experimental unit was considered as the tank rather than the individual fish to avoid pseudoreplication.

### 2.2. Inoculum Preparation and Infection

The *P. vermicola* bacterial strain used in this study was previously isolated and identified in our laboratory [9] from aseptically collected skin lesions and internal organs (liver, spleen, and heart) of Nile tilapia obtained from aquaculture farms in Chiapas, Mexico, where the fish exhibited clinical signs of disease. Bacterial cultivation and colony-forming unit (CFU) determination were performed following the protocol described [18]. For the experimental challenge, tilapia were first anesthetized and then intraperitoneally injected with 0.2 mL of a bacterial suspension containing 1 × 10^7^ CFU/mL of *P. vermicola*. The inoculum concentration was determined based on the previously established median lethal dose (LD_50_). The LD_50_ was estimated from preliminary infection assays conducted under similar experimental conditions. Fish were intraperitoneally injected with graded doses of *P. vermicola* (1 × 10^7^, 1 × 10^8^ and 1 × 10^9^ CFU/fish), along with a saline-injected control group and monitored for seven days. Mortality data were transformed to probit values and plotted against the log_10_ of the bacterial dose. A linear regression model was fitted to estimate the DL_50_. The control group received 0.2 mL of sterile saline solution. After inoculation, fish were maintained under the same experimental conditions for seven days post-infection.

### 2.3. DNA Extraction and Sequencing

At the end of the trial, fish were fasted for 24 h prior to sampling. Fish were euthanized by an overdose of tricaine methanesulfonate (MS-222; 250 mg/L) added directly to the aquarium. The intestinal tract was subsequently dissected, and intestinal contents were collected using the scraping technique. Samples were placed in sterile tubes, and intestinal contents from all of the surviving fish in each aquarium were pooled to generate a single representative pooled sample per experimental replicate (*n* = 3 pools per treatment). 250 mg of pooled intestinal content was used for DNA extraction. Samples were homogenized using a FastPrep-5G system (MP Biomedicals, Santa Ana, CA, USA) at 6.0 m/s for 40 s. Following homogenization, samples were centrifuged at 14,000× *g* for 5 min.

Total microbial DNA was extracted using the FastDNA™ Spin Kit (MP Biomedicals, Santa Ana, CA, USA) for Soil according to the manufacturer’s instructions. Library preparation and sequencing were performed at the Arizona Genetics Core at the University of Arizona. For bacterial identification, the V3–V4 region of the 16S rRNA gene was amplified using the primers 347F and 803R [19], whereas the V9 hypervariable region of the 18S rRNA gene was amplified with the primers 1391f and EukBr [20] to characterize the intestinal eukaryotic microbiota. Amplicon libraries were sequenced on an Illumina MiSeq platform using a 2 × 250 bp paired-end configuration. The raw sequence reads were deposited into the NCBI Sequence Read Archive (SRA) database (accession number PRJNA1442838).

### 2.4. Data Processing

Raw sequencing data were imported into QIIME 2 (version 2025.7; QIIME 2 Development Team, University of Colorado, Boulder, Boulder, CO, USA) and demultiplexed. Paired-end reads were subsequently subjected to quality control, error correction, paired-end merging, and chimera removal using the DADA2 algorithm [21]. During this process, reads were trimmed at the initial positions to remove low-quality regions, using marker-specific truncation and filtering parameters. Detailed DADA2 processing parameters and sequence filtering criteria are provided in Supplementary Methods S2, while per-sample sequencing statistics are available in Supplementary Methods S3.

Taxonomic assignment of ASVs was performed using Naive Bayes classifiers trained on the SILVA v138 database [22], filtered by domain according to the analyzed marker (Bacteria for 16S rRNA and Eukaryota for 18S rRNA). Classifiers were trained explicitly for the V3–V4 (16S rRNA) and V9 (18S rRNA) regions, respectively. Seven taxonomic ranks were used to generate relative abundance bar plots.

Alpha diversity (Shannon and Simpson indices) and beta diversity analyses (Bray–Curtis, Jaccard, and UniFrac (distance metrics) were performed using MicrobiomeAnalyst 2.0 [23]. Differences among treatments were evaluated using one-way ANOVA (*p* < 0.05), and beta diversity was visualized through principal coordinates analysis (PCoA).

Differences in microbial community composition between treatments were assessed using PERMANOVA and MiRKAT analyses implemented in MicrobiomeAnalyst 2.0 [23]. Differential abundance of individual taxa was evaluated using DESeq2 as implemented in MicrobiomeAnalyst 2.0 [23]. A Pearson correlation analysis was conducted, and the microbial dysbiosis index (MD-index) was calculated as the logarithm of the ratio between the total abundance of taxa increased and decreased in infected fish relative to the control group [18].

The functional potential was predicted from 16S rRNA gene amplicon data using PICRUSt2 (v2.5.0) [24]. ASVs were placed into a reference phylogeny, and Enzyme Commission (EC) family abundances were inferred using hidden-state prediction. Predicted EC abundances, normalized by predicted 16S rRNA gene copy number, were used for metagenome inference and subsequent reconstruction of MetaCyc pathways [25] via the MinPath procedure implemented in PICRUSt2. Unstratified pathway abundance tables were used for downstream analyses, including LEfSe [26] for differential pathway enrichment between control and *P. vermicola*-infected fish (Kruskal–Wallis, α = 0.05; LDA > 2.0). Additional information of processing are in Supplementary Methods S4.

## 3. Results

### 3.1. Survival Rate

Infection with *P. vermicola* resulted in a pronounced reduction in survival after seven days, with infected fish exhibiting a survival rate of 33.33 ± 7.2%, whereas no mortality was observed in the control group (Table 1).

### 3.2. Composition of the Bacterial Intestinal Microbiota

**Figure 1:**
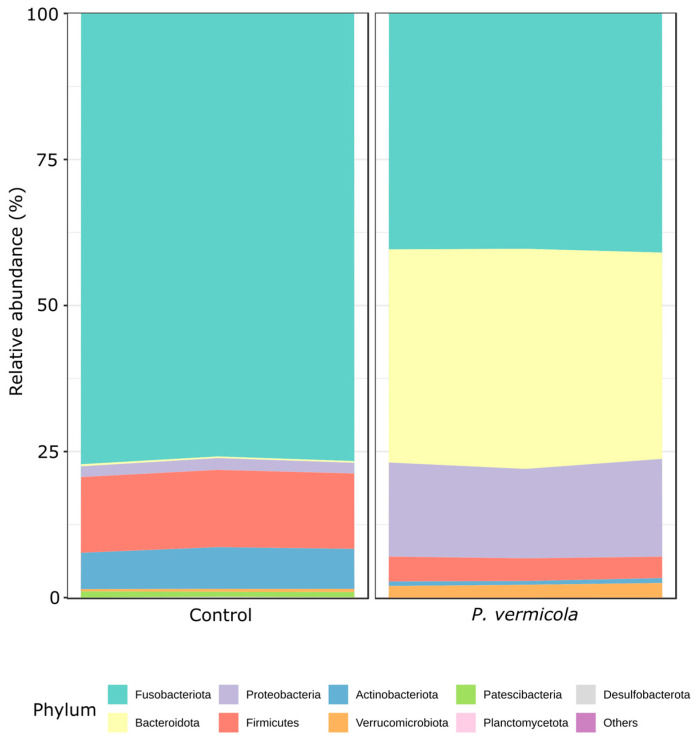
Mean relative abundance at the phylum level of the bacterial intestinal microbiota in control Nile tilapia and fish infected with *P. vermicola* (*n* = 3 pooled samples per treatment).

Analysis of bacterial composition at the phylum level (Figure 1) revealed that *P. vermicola* infection exerted a clear and consistent impact on the structure of the intestinal microbiota. Although Fusobacteriota primarily dominated the intestinal communities in both groups (76.55 ± 0.67% in controls vs. 40.54 ± 0.35% in infected fish), infected fish exhibited a clear compositional shift, characterized by a markedly higher representation of Bacteroidota, which rose from 0.30 ± 0.27% in controls to 36.49 ± 1.19% in infected fish. Overall, phyla detected across both treatments included Actinobacteriota, Bacteroidota, Desulfobacterota, Firmicutes, Fusobacteriota, Planctomycetota, Proteobacteria, and Verrucomicrobiota. Notably, the phylum Patescibacteria was not detected above the filtering threshold in fish infected with *P. vermicola*.

Consistent with this community-wide restructuring, infected fish showed a significant enrichment of Bacteroidota and Proteobacteria (DESeq2, *p* < 0.05) relative to the control group. In contrast, Fusobacteriota and Patescibacteria exhibited significant reductions in relative abundance in infected fish (*p* < 0.05), while remaining comparatively enriched in healthy individuals. Other phyla, including Firmicutes and Actinobacteriota, were detected in both groups but showed lower relative abundances in infected fish, further supporting the notion that *P. vermicola* infection induces a broad, non-random remodeling of the intestinal microbiota rather than isolated taxonomic fluctuations.

**Figure 2:**
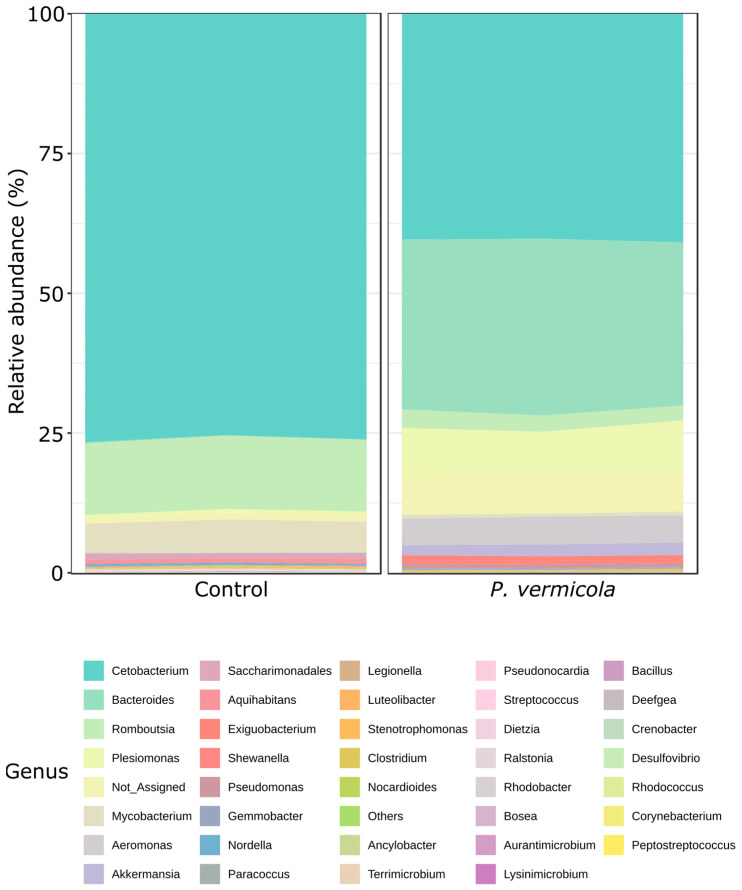
Mean relative abundance at the genus level of the bacterial intestinal microbiota in control Nile tilapia and fish infected with *P. vermicola* (*n* = 3 pooled samples per treatment).

Changes were also detected at the genus level (Figure 2); significant increases in the differential abundance of *Aeromonas*, *Shewanella*, *Plesiomonas*, and *Pseudomonas* were observed in fish infected with *P. vermicola* compared with the control group (DESeq2, *p* < 0.05).

#### 3.2.1. Alpha Diversity of the Bacterial Intestinal Microbiota

**Figure 3:**
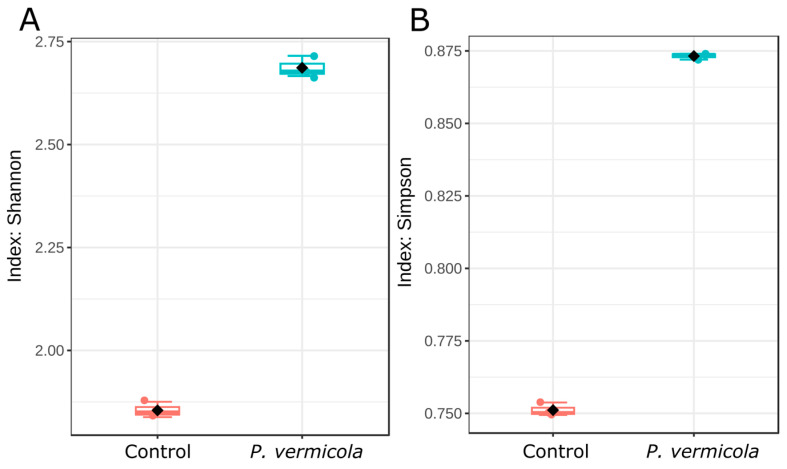
Alpha diversity boxplots based on Shannon (**A**) and Simpson (**B**) indices calculated at the ASV level for the bacterial intestinal microbiota in control Nile tilapia and fish infected with *P. vermicola*. Significant differences were observed for both Shannon and Simpson indices (*p* < 0.05).

The alpha diversity of the intestinal bacterial microbiota is shown in Figure 3. The Shannon index, reflecting both taxonomic richness and evenness, and the Simpson index, emphasizing community dominance and evenness, were significantly higher in fish infected with *P. vermicola* than in the control group (*p* < 0.05).

#### 3.2.2. Beta Diversity of the Bacterial Intestinal Microbiota

**Figure 4:**
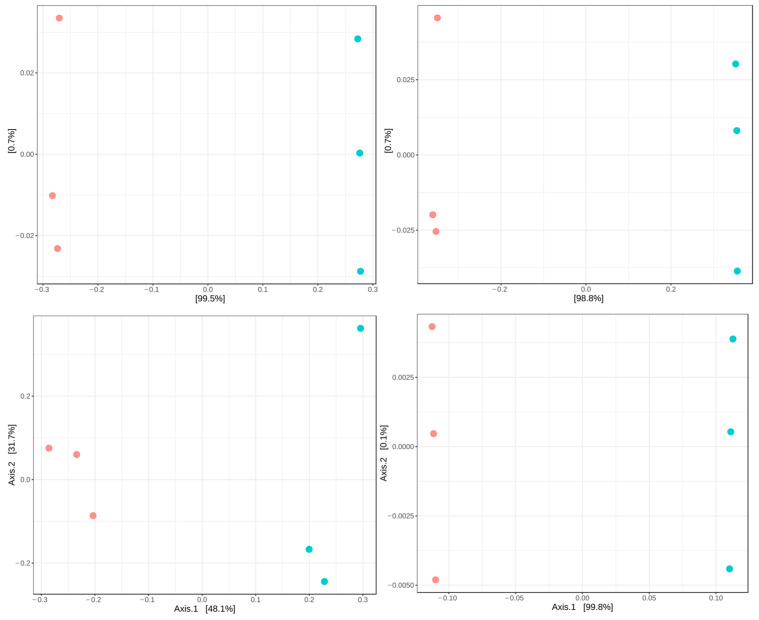
PCoA of the intestinal bacterial microbiota based on Bray–Curtis (A), Jaccard (B), unweighted UniFrac (C), and weighted UniFrac (D) distance metrics calculated at the ASV level in control Nile tilapia and fish infected with *P. vermicola*. Red: control. Blue: infection treatment.

PCoA plots based on Jaccard (presence–absence), Bray–Curtis (abundance-based dissimilarity), and UniFrac distances (phylogenetic relatedness) showed a separation trend between control fish and fish infected with *P. vermicola* (Figure 4). Despite this visual distribution, PERMANOVA analysis did not detect significant differences in community structure (*p* > 0.05). In contrast, MiRKAT analysis revealed significant associations between bacterial community composition and infection status for Jaccard, Bray–Curtis, and weighted UniFrac distances (*p* < 0.05).

#### 3.2.3. Correlation Analysis and Dysbiosis Index of the Bacterial Intestinal Microbiota

**Figure 5:**
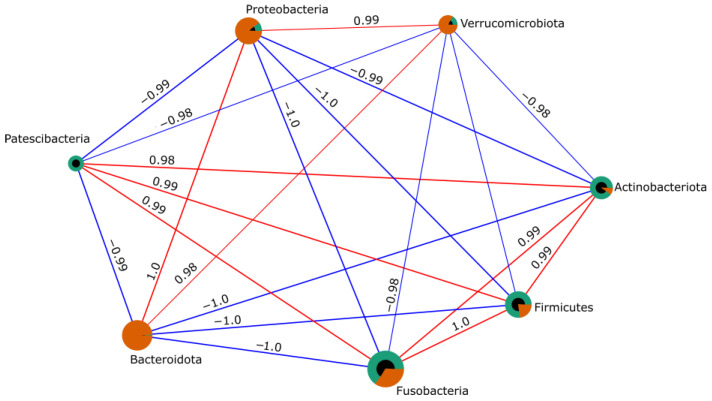
Pearson correlation network (*p* < 0.05) showing significant associations among prokaryotic bacterial phyla of the intestinal microbiota in control Nile tilapia and fish infected with *P. vermicola.* Green: control. Orange: infected treatment. Red lines indicate positive correlations (r > 0), whereas blue lines indicate negative correlations (r < 0).

Pearson correlation analysis revealed multiple significant associations among bacterial phyla (*p* < 0.05), showing both positive and negative correlations among prokaryotic groups (Figure 5). Strong positive correlations were observed among Bacteroidota, Proteobacteria, and Verrucomicrobiota, whereas these phyla showed negative correlations with Fusobacteriota, Firmicutes, Actinobacteriota, and Patescibacteria. The microbial dysbiosis index showed an MD-index value of 3.79 in fish infected with *P. vermicola.*

#### 3.2.4. Metabolic Pathways

**Figure 6:**
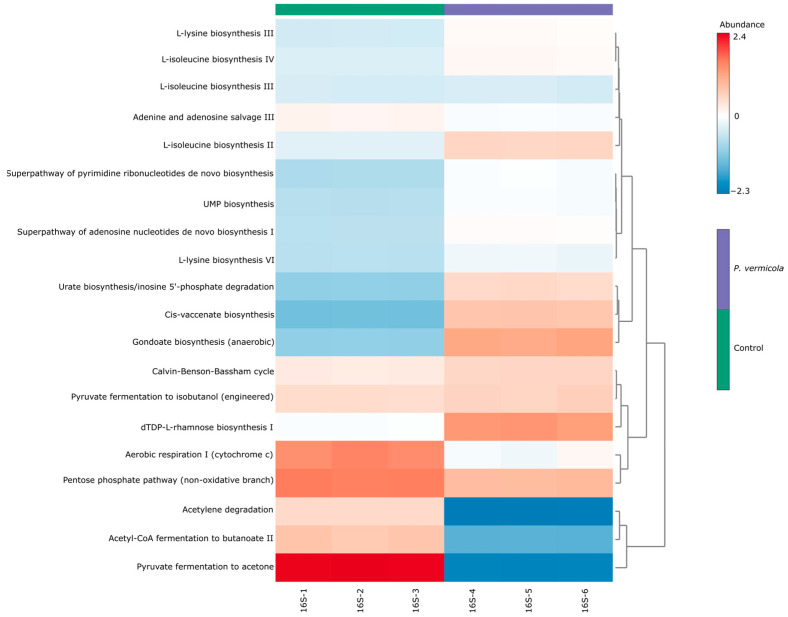
Heatmap showing the predicted abundance of the top 20 MetaCyc pathways. The color gradients of the different color blocks represent changes in the abundances of different functions across the treatments.

The heatmap of the 20 MetaCyc pathways with the highest global mean abundance (Figure 6) revealed differences in functional profiles between the experimental groups. These patterns reflect differences in the predicted abundance of the predominant metabolic pathways between the two groups.

**Figure 7:**
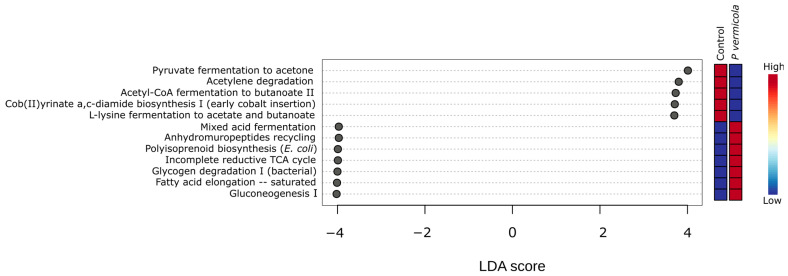
Differentially abundant MetaCyc pathways between control and *P. vermicola*-infected fish identified by LEfSe analysis. Each dot represents a metabolic pathway, and the LDA score indicates the effect size of differential abundance. Only pathways with an absolute LDA score ≥ 2 are shown.

Linear discriminant analysis effect size (LEfSe) (Figure 7) revealed significant differences in predicted MetaCyc pathway abundances between control and *P. vermicola*-infected fish (LDA score > 2.0; *p* < 0.05). Control samples showed a significantly higher relative abundance of the pathways: pyruvate fermentation to acetone, acetylene degradation, acetyl-CoA fermentation to butanoate II, cob(II)yrinate a,c-diamide biosynthesis I (early cobalt insertion), and L-lysine fermentation to acetate and butanoate. In contrast, infected fish exhibited significantly increased relative abundances of multiple pathways, including mixed acid fermentation, gluconeogenesis I, glycogen degradation I (bacterial), fatty acid elongation–saturated, polyisoprenoid biosynthesis (*E. coli*), anhydromuropeptides recycling, and incomplete reductive TCA cycle. These pathways consistently contributed to discrimination between treatments based on LDA scores and relative abundance distributions across samples.

### 3.3. Composition of the Intestinal Microeukaryotic Microbiota

**Figure 8:**
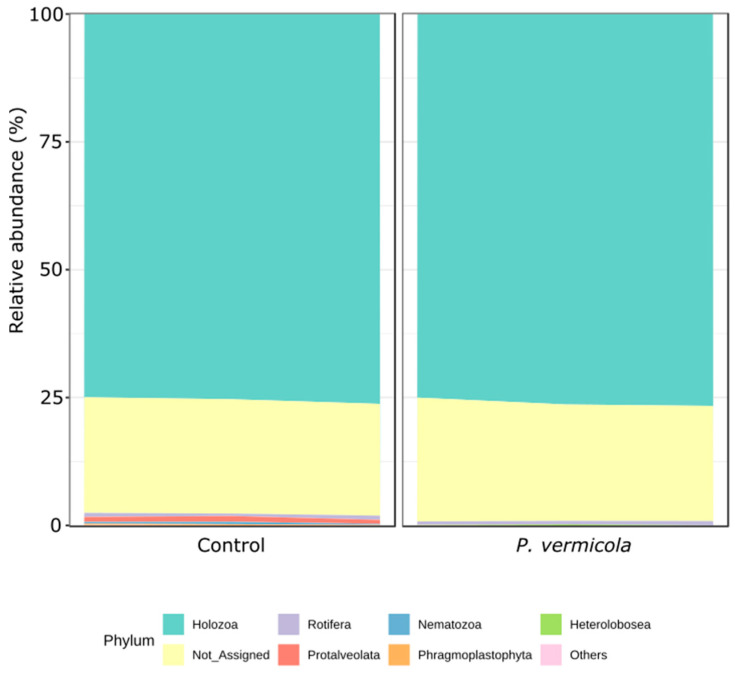
Mean relative abundance at the phylum level of the intestinal microeukaryotic microbiota in control Nile tilapia and fish infected with *P. vermicola* (*n* = 3 pooled samples per treatment).

The analysis of microeukaryotic composition at the phylum level (Figure 8) showed that intestinal communities were primarily dominated by Holozoa, which displayed the highest relative abundance. Phragmoplastophyta, Nematozoa, and Protalveolata exhibited lower differential abundances in fish infected with *P. vermicola* compared with the control group (DESeq2, *p* < 0.05).

#### 3.3.1. Alpha Diversity of the Intestinal Microeukaryotic Microbiota

**Figure 9:**
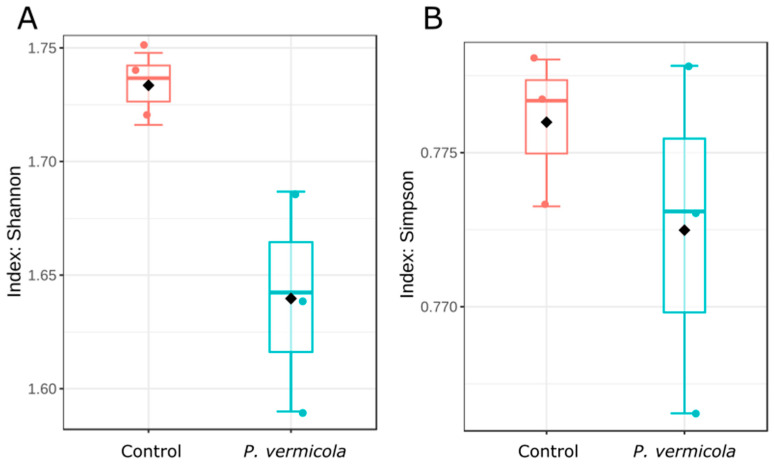
Alpha diversity boxplots based on Shannon (**A**) and Simpson (**B**) indices calculated at the ASV level for the intestinal microeukaryotic microbiota in control Nile tilapia and fish infected with *P. vermicola*. Significant differences in the Shannon index were observed between treatments (*p* < 0.05).

The alpha diversity of the intestinal microeukaryotic microbiota is shown in Figure 9. The Shannon index was significantly higher in the control group than in fish infected with *P. vermicola* (*p* < 0.05), whereas the Simpson index did not differ between treatments (*p* > 0.05).

#### 3.3.2. Beta Diversity of the Intestinal Microeukaryotic Microbiota

**Figure 10:**
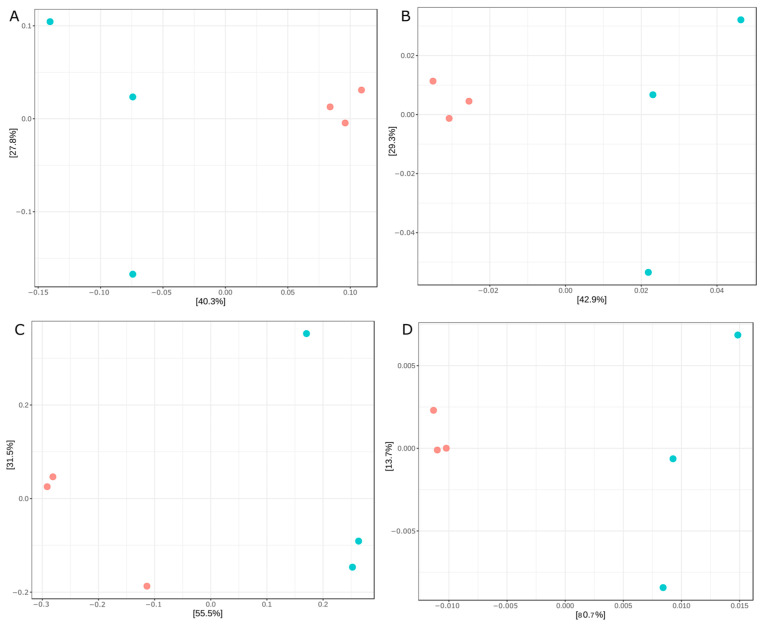
PCoA of the intestinal microeukaryotic microbiota based on Jaccard (**A**), Bray–Curtis (**B**), unweighted UniFrac (**C**), and weighted UniFrac (**D**) distance metrics calculated at the ASV level in control Nile tilapia and fish infected with *P. vermicola*. Red: control. Blue: infection treatment.

PCoA plots based on Jaccard (a), Bray–Curtis (b), unweighted UniFrac (c), and weighted UniFrac (d) distances, as shown in Figure 10, revealed a separation trend between the control group and fish infected with *P. vermicola*. However, PERMANOVA analysis did not detect significant differences in community structure (*p* > 0.05). In contrast, MiRKAT analysis revealed significant associations between microeukaryotic community composition and infection status for Jaccard and Bray–Curtis distances (*p* < 0.05).

#### 3.3.3. Correlation Analysis and Dysbiosis Index of the Intestinal Microeukaryotic Microbiota

**Figure 11:**
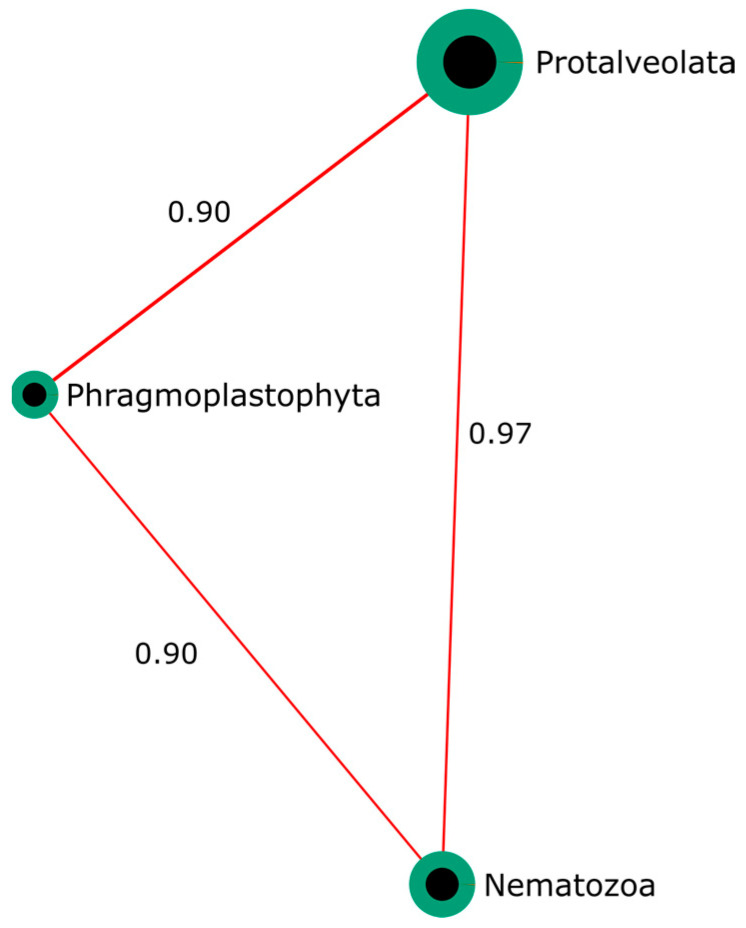
Pearson correlation network (*p* < 0.05) showing significant associations among eukaryotic phyla of the intestinal microbiota. Green: control. Orange: infection treatment. Red lines indicate positive correlations (r > 0).

Pearson correlation analysis revealed significant positive associations among Nematozoa, Phragmoplastophyta, and Protalveolata (*p* < 0.05) as shown in Figure 11, with high correlation coefficients (r = 0.89–0.97), indicating consistent co-occurrence among these microeukaryotic phyla within the intestinal microbiota. The MD-index could not be calculated because no significantly depleted taxa were identified.

## 4. Discussion

This study evaluated changes in the composition of prokaryotic and microeukaryotic communities within the intestinal microbiota of tilapia, integrating both components. Most previous studies have primarily focused on the bacterial fraction; however, the fish microbiota comprises a wide diversity of microorganisms, including archaea and eukaryotes, whose ecological roles remain poorly characterized [27,28]. In this context, the simultaneous analysis of bacterial and eukaryotic communities expands the understanding of intestinal microbiota responses to infectious processes.

The composition of intestinal microorganisms in fish is influenced by multiple environmental, biological, and behavioral factors that modulate community stability [10,29]. Among these, pathogenic infections are among the main drivers of shifts in intestinal microbial structure, often associated with changes in specific host-associated bacterial taxa [30]. Such alterations may promote the proliferation of opportunistic bacteria and contribute to microbial imbalance in aquaculture systems [31,32,33].

Infectious processes have been linked to states of intestinal dysbiosis characterized by the expansion of opportunistic microorganisms, disruption of epithelial integrity, and modulation of host immune responses. These changes can increase intestinal permeability and susceptibility to systemic inflammatory processes, reflecting a strong relationship between intestinal microbiota dynamics and fish health [34].

In the present study, *P. vermicola* infection was associated with changes in the structure of tilapia’s prokaryotic intestinal microbiota. Alpha diversity indices showed higher values in infected fish, suggesting a community reorganization characterized by increased microbial heterogeneity. This pattern has been previously described in scenarios of intestinal disturbance, where changes in alpha diversity may reflect community restructuring associated with the incorporation of opportunistic taxa [35,36]. In terms of community structure, although PERMANOVA did not detect significant differences, MiRKAT analysis revealed significant associations, accompanied by an elevated dysbiosis index (MD-index = 3.79), suggesting that infection-related shifts may involve subtle yet structured compositional changes that are not fully captured by permutation-based methods under limited replication. In contrast, another study [18] did not detect significant differences in alpha or beta diversity between infected-with-*P. vermicola* and control groups, and ordination analyses showed no clear separation between treatments. These differences may be explained by methodological variations between studies, including the use of different alpha diversity metrics (Chao1 and Fisher indices vs. Shannon and Simpson), as well as the application of more sensitive statistical approaches in the present study, such as MiRKAT, which are capable of detecting subtle compositional shifts not captured by PERMANOVA alone. Therefore, our results suggest that *P. vermicola* infection may induce structured changes in the intestinal microbiota that require integrative and sensitive analytical frameworks to be properly detected. At the taxonomic level, a decrease in Fusobacteriota and Patescibacteria was observed in fish infected with *P. vermicola* compared with healthy fish. Fusobacteriota was one of the dominant phyla in the analyzed tilapia, and the genus *Cetobacterium* showed a reduction in infected fish. This result is consistent with previous reports describing similar decreases of *Cetobacterium* in diseased fish [37]. Several studies have associated this genus with metabolic functions relevant to intestinal homeostasis, including vitamin B12 synthesis and participation in metabolic pathways related to host nutrition [38,39,40,41], suggesting that its reduction could be linked to functional changes within the intestinal ecosystem.

An increase in Bacteroidota and Proteobacteria was also detected in infected fish compared with healthy individuals, a pattern previously reported in the intestinal microbiota of infected fish. Previous studies have reported marked increases in Proteobacteria in diseased organisms; for example, a study observed a higher relative abundance of this phylum in diseased fish (53.4%) compared with healthy fish (18.9%) [36]. Another study reported similar trends, with Proteobacteria reaching 86.0% in diseased fish versus 55.0% in healthy organisms [42]. Several studies have demonstrated that host inflammatory responses can favor the expansion of facultative anaerobic bacteria within the intestinal lumen, particularly members of the family Enterobacteriaceae, reflected compositionally by an increase in the phylum Proteobacteria [43,44,45]. During inflammatory processes, the generation of electron acceptors derived from host metabolism provides competitive advantages to these taxa, promoting their proliferation within altered microbial communities [46]. In this context, the simultaneous increase in Proteobacteria and Enterobacteriaceae observed in the present study may reflect a compositional shift characterized by the expansion of facultative anaerobic Enterobacteriaceae, accompanied by a reduction in dominant lineages associated with more stable intestinal states. This shift may also reflect the disruption of intestinal homeostasis, where opportunistic taxa expand under host stress and immune activation, potentially altering redox balance and nutrient availability within the gut environment. Together, these compositional changes indicate a transition from a functionally integrated intestinal ecosystem toward a disturbance-adapted configuration. The reduction of *Cetobacterium* and other taxa associated with nutrient exchange and host metabolic support, combined with the expansion of facultative anaerobic Proteobacteria, is consistent with a shift toward a dysbiotic state in which microbial interactions are reorganized under inflammation-driven redox conditions. Rather than representing isolated taxonomic fluctuations, this pattern reflects a coordinated ecological restructuring of the intestinal microbiota in response to pathogen challenge.

Among the genera that showed higher abundance in infected fish were *Aeromonas*, *Pseudomonas*, *Plesiomonas*, and *Shewanella*, taxa frequently associated with infectious processes in aquaculture systems [47,48]. Several studies have described *Aeromonas* as an opportunistic pathogen capable of acting as a secondary invader in immunocompromised fish [49], whereas *Pseudomonas* is commonly found in aquatic environments and as part of the intestinal microbiota, but may proliferate under ecological imbalance [50]. Likewise, *Plesiomonas shigelloides* has been reported as an opportunistic species in freshwater fish, including tilapia [51], and species of the genus *Shewanella* have recently been described as emerging bacteria associated with stress and increased susceptibility to infections in aquaculture environments [52]. In this context, the increase of these genera in infected fish may be interpreted as an expansion of opportunistic microorganisms within an altered intestinal community.

LEfSe analysis revealed that infected fish exhibited enrichment of multiple pathways associated with central carbon metabolism, including mixed-acid fermentation, gluconeogenesis I, and glycogen degradation I. Together, these pathways enhance metabolic flexibility by allowing bacteria to balance redox demands, generate glucose from non-carbohydrate substrates, and mobilize internal carbon reserves under fluctuating nutrient conditions. Similar metabolic reconfigurations have been described in intestinal infection models, where fermentative and gluconeogenic capacities contribute to bacterial adaptation and colonization fitness [53,54]. The combined enrichment of these pathways in *P. vermicola*-infected fish suggests a coordinated shift in microbial carbon metabolism compatible with infection-associated ecological perturbation, although direct metabolic measurements would be required to confirm these mechanisms.

The effects of infection on the intestinal microeukaryotic community were partially supported by the data. Differences in alpha diversity, as measured by the Shannon index, were observed, suggesting changes in community structure. In addition, MiRKAT analysis revealed significant associations, suggesting structured compositional shifts. However, PERMANOVA did not detect significant differences; therefore, these patterns should be interpreted with caution given the limited sample size. Among the microeukaryotic phyla affected by *P. vermicola*, notable decreases were observed in Protalveolata, Nematozoa, and Phragmoplastophyta. Alveolates have been previously reported in tilapia and in fertilized aquaculture systems, where they are commonly associated with microeukaryotic assemblages originating from natural productivity driven by feed inputs and the decomposition of organic waste [55,56]. In this scenario, the detected alveolate lineages may represent ecological components linked to microbial trophic networks and the environmental dynamics of the culture system. Therefore, their decrease in infected fish could be associated with changes in the structure of the intestinal microeukaryotic community.

The phylum Nematozoa also showed a significant decrease in fish infected with *P. vermicola*. This group includes both free-living organisms and parasitic forms. In aquatic ecosystems, nematodes are part of microscopic trophic networks associated with detritus and have been described as sensitive indicators of ecological changes and environmental conditions [57]. The observed reduction in Nematozoa could be interpreted as a decrease in the trophic complexity of the intestinal microbiota’s microeukaryotic fraction, possibly associated with modifications in the ecological niche during infection. This pattern is consistent with a transition toward intestinal communities dominated by facultative opportunistic bacteria and a simultaneous reduction of microeukaryotic groups linked to trophic networks.

Pearson correlation analysis revealed significant positive associations among the microeukaryotic phyla Protalveolata, Nematozoa, and Phragmoplastophyta (*p* < 0.05), suggesting a compositional co-occurrence pattern within the intestinal microeukaryotic community. Co-occurrence networks in aquatic microbiotas have been widely used to infer ecological structuring and shared environmental niches, where positive correlations often reflect taxa responding similarly to trophic resources or habitat conditions [58]. In aquaculture ecosystems, bipartite network analyses have demonstrated that microeukaryotes frequently form modular structures linked to biofilms, particulate organic matter, and nutrient pathways, indicating that groups with strong positive correlations may represent ecological modules [59]. In this context, the reduction of Protalveolata, Nematozoa, and Phragmoplastophyta in infected fish may indicate the disruption of a previously co-structured microeukaryotic assemblage associated with environmental inputs such as feed particles, detrital material, or biofilm-derived communities. Similar studies in aquatic microbial systems have shown that loss of microeukaryotic taxa can destabilize co-occurrence networks and reduce the complexity of trophic interactions within microbial ecosystems [59]. However, it is important to consider that the infection model used in this study, based on intraperitoneal injection, allows precise control over the infectious dose and experimental reproducibility but bypasses the host’s natural mucosal barriers. Given that mucosa plays a key role in host microbiota interactions [60], this approach may not fully reflect the processes occurring during natural infections. Consequently, the observed changes in microbial communities, both bacterial and microeukaryotic, may not fully represent those arising from environmentally acquired infections, and should therefore be interpreted within the context of this experimental limitation.

Water temperature should also be considered when interpreting the microbiota response observed in this study. Although 20 °C is within the tolerated thermal range of Nile tilapia [61], it is lower than the temperature commonly associated with routine tilapia culture [62] and differs from the thermal condition under which the *P. vermicola* isolate was originally obtained. Therefore, this experimental condition may have influenced host physiology, immune responses, and gut microbial dynamics, potentially modulating the response to infection. Nevertheless, the bacterial concentration used in the present study was based on a previous LD_50_ assessment conducted at 20 °C, supporting the use of this challenge model under the selected temperature condition. Even so, this difference should be considered when comparing the present results with studies performed at higher temperatures or with natural infections scenarios. Future studies conducted at temperatures closer to standard tilapia culture conditions would help improve comparability and further clarify temperature-dependent host-microbiota pathogen interactions.

Overall, the observed changes in bacterial and microeukaryotic communities suggest that *P. vermicola* infection may be associated with a reorganization of the tilapia intestinal microbiota, characterized by the expansion of opportunistic taxa and the simultaneous reduction in taxa potentially linked to more stable ecological states. This type of alteration reflects shifts in the ecological balance of the microbiota, in which environmental or pathological perturbations can modify microbial structure and favor the dominance of microorganisms with greater tolerance to altered conditions [31,63].

Finally, although the characterization of the intestinal microeukaryotic microbiota was not a primary objective of this study, the data provides a preliminary descriptive snapshot of eukaryotic taxa present in healthy Nile tilapia. The control group was characterized mainly by the presence of Holozoa, together with Protalveolata, Phragmoplastophyta, and Nematozoa, which have previously been reported in fish-associated and aquaculture environments. While these observations are strictly descriptive, they indicate that a detectable microeukaryotic component is present in the tilapia intestine under non-infected conditions.

When interpreted together with the prokaryotic patterns observed in infected fish, the reduction in these microeukaryotic groups suggests that pathogen-driven disturbance is not restricted to bacterial communities but extends to the entire intestinal microbial network. These changes support the view of the gut microbiota as a multi-kingdom ecological system in which cross-domain interactions contribute to community stability. The simultaneous alteration of bacterial and eukaryotic components indicates that infection may disrupt trophic relationships, nutrient exchange, and microhabitat structure within the intestine, reinforcing the need to move beyond a bacteria-centered approach in fish microbiota research.

## 5. Conclusions

This study suggests that pathogen challenge in tilapia is associated with coordinated changes in the intestinal microbiota across multiple microbial kingdoms rather than as isolated bacterial shifts, providing experimental evidence that infection-driven dysbiosis must be interpreted at the ecosystem level. The coordinated changes observed across different microbial kingdoms indicate that infection-related perturbations could alter the balance of the intestinal ecosystem beyond isolated taxonomic effects, potentially influencing community stability and ecological interactions. These findings highlight the importance of considering multi-kingdom perspectives when evaluating microbiota responses in aquaculture species.

## Figures and Tables

**Table 1:** Survival rate of Nile tilapia (*Oreochromis niloticus*) after experimental infection with *P. vermicola*.

Treatment	Survival Rate (%)
Control	100 ± 0.00
*P. vermicola*	33.33 ± 7.22

## Data Availability

The data presented in this study are available on request from the corresponding author.

## Supplementary Materials

The following supporting information can be downloaded at: https://www.mdpi.com/article/10.3390/ani16081180/s1. Supplementary Methods S1: Water quality.; Supplementary Methods S2: DADA2 processing parameters and sequence filtering criteria.; Supplementary Methods S3: Sequencing data summary for 16S and 18S rRNA gene datasets.; Supplementary Methods S4: Functional prediction using PICRUSt2.
