# Supplementary material for: Providencia vermicola Infection Alters Bacterial and Microeukaryotic Gut Community Composition in Nile Tilapia
Source: Animals (Basel). 2026 Apr 12;16(8):1180. doi: 10.3390/ani16081180 (PMC13113470; doi:10.3390/ani16081180)
Supplement: Supplementary file 1 [file animals-16-01180-s001.zip › animals-4198420-supplementary.pdf]

Supplementary Material

Supplementary Methods S1. Water quality

Table S1. Water quality parameters measured in each experimental treatment.

Treatment	NH_4^+ (mg/L)	NO_2^- (mg/L)	NO_3^- (mg/L)
Control	0.28 ± 0.21	0.68 ± 0.53	0.21 ± 0.16
P. vermicola	0.31 ± 0.22	0.83 ± 0.67	0.26 ± 0.18

Supplementary Methods S2. DADA2 processing parameters and sequence filtering criteria

Paired-end reads were processed using the DADA2 plugin in QIIME 2. For the 16S rRNA gene (V3–V4 region), forward and reverse reads were trimmed by 20 bp at the 5′ ends and truncated at 245 bp, while for the 18S rRNA gene (V9 region), reads were trimmed by 10 bp and truncated at 200 bp based on quality score profiles. Reads containing ambiguous bases were discarded (max-n = 0), and all other parameters were set to default values. For diversity analyses, data were rarefied to an even sequencing depth of 23,206 reads per sample for the 16S dataset and 3,828 reads per sample for the 18S dataset.

Supplementary Methods S3. Sequencing data summary for 16S and 18S rRNA gene datasets

A total of 25,780 to 38,353 raw reads per sample were obtained for the 16S rRNA gene sequencing. After quality filtering and denoising using DADA2, between 23,259 and 34,725 reads per sample were retained, representing 89.31–91.66% of the initial sequences. Following read merging and chimera removal, the number of high-quality non-chimeric reads ranged from 23,206 to 34,307 per sample (78.44–90.22% of the input reads). In total, 253 amplicon sequence variants (ASVs) were initially identified, of which 162 remained after post-processing and filtering steps.

A total of 33,982 to 52,189 raw reads per sample were obtained for the 18S rRNA gene sequencing. After quality filtering and denoising using DADA2, between 31,536 and 48,408 reads per sample were retained, representing 91.41–93.98% of the initial sequences. Following read merging and chimera removal, the number of high-quality non-chimeric reads ranged from 22,566 to 34,521 per sample (65.15–66.93% of the input reads). In total, 111 amplicon sequence variants (ASVs) were initially identified, of which 80 remained after post-processing and filtering steps.

Supplementary Methods S4. Functional prediction using PICRUSt2

Functional prediction was performed using PICRUSt2 (v2.5.0) based on 16S rRNA gene amplicon data. Amplicon sequence variants (ASVs) and feature tables generated in QIIME2 were exported in FASTA and BIOM formats and used as input for the analysis.

ASV sequences were placed into a reference phylogeny using the place_seqs.py script with default parameters. Hidden-state prediction (HSP) was performed using hsp.py to estimate 16S rRNA gene copy number and to infer Enzyme Commission (EC) family abundances.

Metagenome prediction was conducted using metagenome_pipeline.py, which normalizes EC abundances by predicted 16S rRNA gene copy number and weights them according to ASV abundance across samples. Both stratified and unstratified outputs were generated.

Pathway inference was performed using the pathway_pipeline.py script, which applies the MinPath algorithm to reconstruct MetaCyc pathways from EC abundances. Functional annotations were assigned using the MetaCyc mapping file included in PICRUSt2 (metacyc_pathways_info.txt.gz).

Unstratified pathway abundance tables were used for downstream analyses. Differential pathway enrichment between control and P. vermicola-infected fish was assessed using LEfSe, applying the Kruskal–Wallis test (α = 0.05) and a logarithmic LDA score threshold of 2.0.

The following parameters were used during the analysis: default settings for place_seqs.py and hsp.py; --strat_out enabled in metagenome_pipeline.py; and --per_sequence_contrib enabled in pathway_pipeline.py. All analyses were conducted using default parameters unless otherwise specified.
